# The proposed new species, cacao red vein virus, and three previously recognized badnavirus species are associated with cacao swollen shoot disease

**DOI:** 10.1186/s12985-017-0866-6

**Published:** 2017-10-19

**Authors:** Nomatter Chingandu, Koffie Kouakou, Romain Aka, George Ameyaw, Osman A. Gutierrez, Hans-Werner Herrmann, Judith K. Brown

**Affiliations:** 1 School of Plant Sciences, University of Arizona, Tucson, AZ 85721 USA; 2 Centre National de Recherche Agronomique (CNRA), Programme Cacao, Divo, Côte d’Ivoire; 3 Cocoa Research Institute of Ghana, New Tafo-Akim, Ghana; 4 USDA-ARS Subtropical Horticultural Research Station, Miami, FL 33158 USA

**Keywords:** *Caulimoviridae*, Cacao virus, Double-stranded DNA plant virus, Mealybug-transmitted virus, Pararetrovirus

## Abstract

**Background:**

*Cacao swollen shoot virus* (CSSV), *Cacao swollen shoot CD virus* (CSSCDV), and *Cacao swollen shoot Togo A virus* (CSSTAV) cause cacao swollen shoot disease (CSSD) in West Africa. During 2000–2003, leaf and shoot-swelling symptoms and rapid tree death were observed in cacao in Cote d’Ivoire and Ghana. Molecular tests showed positive infection in only ~50–60% of symptomatic trees, suggesting the possible emergence of an unknown badnavirus.

**Methods:**

The DNA virome was determined from symptomatic cacao samples using Illumina-Hi Seq, and sequence accuracy was verified by Sanger sequencing. The resultant 14, and seven previously known, full-length badnaviral genomic and RT-RNase H sequences were analyzed by pairwise distance analysis to resolve species relationships, and by Maximum likelihood (ML) to reconstruct phylogenetic relationships. The viral coding and non-coding sequences, genome organization, and predicted conserved protein domains (CPDs) were identified and characterized at the species level.

**Results:**

The 21 CSSD-badnaviral genomes and RT-RNase H sequences shared 70–100% and 72–100% identity, respectively. The RT-RNase H analysis predicted four species, based on an ≥80% species cutoff. The ML genome sequence tree resolved three well-supported clades, with ≥70% bootstrap, whereas, the RT-RNase H phylogeny was poorly resolved, however, both trees grouped CSSD isolates within one large clade, including the newly discovered Cacao red vein virus (CRVV) proposed species. The genome arrangement of the four species consists of four, five, or six predicted open reading frames (ORFs), and the CPDs have similar architectures. By comparison, two New World cacao-infecting badnaviruses encode four ORFs, and harbor CPDs like the West African species.

**Conclusions:**

Three previously recognized West African cacao-infecting badnaviral species were identified, and a fourth, previously unidentified species, CRVV, is described for the first time. The CRVV is a suspect causal agent of the rapid decline phenotype, however Koch’s Postulates have not been proven. To reconcile viral evolutionary with epidemiology considerations, more detailed information about CSSD-genomic variability is essential. Also, the functional basis for the multiple genome arrangements and subtly distinct CPD architectures among cacao-infecting badnaviruses is poorly understood. New knowledge about functional relationships may help explain the diverse symptomatologies observed in affected cacao trees.

**Electronic supplementary material:**

The online version of this article (10.1186/s12985-017-0866-6) contains supplementary material, which is available to authorized users.

## Background

The *Theobroma cacao* (L.) tree, or cacao, is the source of the chocolate bean. The species is endemic to the Amazon Basin in South America and was introduced into West Africa during the 1880’s [1], where 70% of the world’s bulk cocoa supply is now produced. Soon after commercial cacao plantations were established, virus-like symptoms were reported in cacao trees consisting of foliar vein-banding, swellings on vegetative shoots (chupons), and reduced pod size, pod number, and quality of beans [2, 3]. Three to five years post-symptom development, infected trees decline and eventually die [4, 5]. Graft transmission was reported by Steven in 1936 [3], leading to the hypothesis that the swollen shoot disease was caused by a plant virus, endemic to West Africa, referred to as *Cacao swollen shoot virus* (CSSV) [4, 6]. The virus is transmitted in a semi-persistent manner by 14 mealybug species [7], and is not known to be seed-transmitted in cacao [8]. Efforts to develop virus-resistant cacao genotypes have been largely unsuccessful, and so over time, crop losses have been substantial in Cote d’Ivoire [9, 10], Ghana [11–13], Nigeria [14, 15], Sierra Leone [16], and Togo [17, 18]. Until now, CSSV and two other cacao-infecting virus species, *Cacao swollen shoot CD virus* (CSSCDV), and *Cacao swollen shoot Togo A virus* (CSSTAV), have been associated with cacao swollen shoot disease (CSSD) [19], and are members of the genus *Badnavirus* (family, *Caulimoviridae*) [20–23], also referred to as pararetroviruses.

Badnaviruses have a circular, double-stranded DNA genome 7.0–9.2 kilobase pairs (kbp) in size, encapsidated in a non-enveloped bacilliform particle [22, 23]. Replication occurs by a viral-encoded reverse transcriptase (RT), and proceeds through an RNA intermediate [24]. The three CSSD-associated genomes encode four to six open reading frames (ORFs), referred to as ORFs 1–4, X, and Y [10, 25]. The precise function of the badnaviral 16 kDa ORF1 protein is not known. The ORF2 encodes a 15 kDa protein with DNA and RNA binding activity [26], and ORF3 encodes a ~212 kDa polyprotein containing several domains that are cleaved to release a functional movement protein (MP), coat protein (CP), aspartic protease (AP), and the RT and ribonuclease H (RNase H) proteins. The ORFs 4, X, and Y, which overlap ORF3, encode 95, 13, and 14 kDa proteins, respectively, all of unknown function [10, 27].

During the early 2000’s to the present, characteristic leaf and shoot swelling CSSD symptoms, accompanied by rapid tree decline and death about one year after the occurrence of initial foliar symptoms, have been observed in a portion of the trees growing in commercial plantations located in western Ghana and eastern Cote d’Ivoire [25; authors’ personal observations]. Concurrently, previously reliable serological and several polymerase chain reaction (PCR) amplifications failed to detect virus in 40–60% of samples, despite presence of CSSD-like symptoms [2, 18, 28, 29]. Also, primers designed to amplify a hypervariable region of the viral MP (located on ORF3), referred to as the ‘ORF3A’ primers, reported to distinguish nine CSSD-badnavirus variants, collectively failed to detect virus in 25–50% of samples from Cote d’Ivoire and Ghana [10, 18, 28, 30]. Even though this MP locus is not phylogenetically informative at the species level [20], the region is informative of extensive intraspecific variability. Similarly, in a recent study of 124 field isolates from Cote d’Ivoire, only half of the samples were positive for CSSD badnavirus infection by PCR amplification [31]. The primers were designed based on the seven CSSD-associated genome sequences from Cote d’Ivoire, Ghana and Togo available in GenBank, to direct amplification of CP, MP, RNase H, RT, and non-coding region fragments. While one or more primer pairs amplified badnavirus amplicon(s), confirmed as badnavirus by DNA sequencing, overall ~50% of symptomatic leaf and shoot samples yielded no PCR product, indicating greater than expected genomic variability among CSSD badnaviruses [28]. Until now, there has been no systematic study of the extent of genomic variability of CSSD badnaviruses in West Africa.

The lack of sufficient representative full-length CSSD-associated genome sequences has precluded a reconciliation of evolutionary and epidemiological information required to inform disease management practices. The objective of this study was to better understand the extent of genomic variability of CSSD-badnaviruses in Cote d’Ivoire and Ghana associated with swollen shoot symptoms, including the recently observed ‘rapid decline’ phenotype. Here, a genomic pathology approach was taken utilizing Illumina Hi-Seq DNA sequencing for ‘virome discovery’, and validation by PCR amplification with virus-specific primers and Sanger DNA sequencing. The 14 apparently full-length genomes were characterized with respect to pairwise nucleotide (nt) identity, phylogenetic relationships, genome organization, and conserved protein domain architecture, in relation to the seven previously reported CSSD-badnaviral genome sequences from Ghana, Cote d’Ivoire and Togo.

## Methods

### Plant samples, DNA isolation, and rolling circle amplification

Sixty-five leaf or shoot samples were collected from symptomatic cacao trees in commercial plantations, or at experimental plots located at the Centre National de Recherche Agronomique in Cote d’Ivoire during 2012. An additional thirty-five samples were collected in 2015 from commercial plantations or the historical collection maintained at the Cocoa Research Institute of Ghana. Each sample was placed in a 50-ml screwcap tube containing 100% glycerol, transported to the University of Arizona, and held at 4 °C. Total DNA was isolated from 100 mg of plant tissue from all samples using the cetyltrimethyl ammonium bromide (CTAB) DNA extraction method of Doyle and Doyle [32]. Purified DNA (2 μl) was enriched for circular DNA using the Templiphi rolling circle amplification (RCA) kit (GE Healthcare Bio-Sciences), according to manufacturer’s instructions, with modifications [33, 34].

### Illumina sequencing and assembly of paired-end reads

Illumina paired-end libraries were constructed from purified DNA or from RCA products using the TruSeq PE cluster kit, to obtain a 350 bp mean insert size. The Illumina HiSeq 2500 sequencing run was carried out at the University of Arizona Genetics Core facility (Tucson, AZ). The reads were de-multiplexed, and quality was assessed using FASTQC (https://www.bioinformatics.babraham.ac.uk/projects/fastqc/). The adapters were removed, and reads were trimmed using TRIMMOMATIC v0.32 [35] and assembled de novo using SeqMan NGen software v12 (DNASTAR), with and without plant host sequence filtering. For filtering, the *T. cacao* nuclear genome (host plant) contigs were downloaded from GenBank Accessions CM001879.1, CM001888.1, FR7222157.1, and KE132922.1 [36, 37]. Also used for filtering were the cacao chloroplast genome sequence, NC014676.2 and cotton *Gossypium hirsutum* (L.) (cacao relative) mitochondria genome, JX065074.1.

### Annotation of badnavirus-like genomes

The assembled apparently full-length genome sequences were first annotated using BLAST2GO [38] to confirm their identity as badna-like viruses, and then subjected to BLASTn [39] analysis to determine the top hits (similarity scores) based on all sequences available in the Genbank database. Based on established convention for the genus *Badnavirus*, the first nt of the predicted tRNA^met^ priming site was assigned as the first nt coordinate [40, 41] for each full-length CSSD-badnaviral genome sequence. The predicted ORFs were identified for CSSD genome sequences using the NCBI ORF Finder tool, and the conserved protein domains (CPD) were predicted using the NCBI Conserved Domain Database (CDD) [42]. The predicted CPDs were aligned for comparisons using MUSCLE [43], implemented in CLC Sequence Viewer 7.5.

### Pairwise and phylogenetic analyses of coding regions and complete genome sequences

Genome sequences for 122 badnavirus isolates representing 37 species were downloaded from GenBank and the genome was considered intact or based on the RT-RNase H region used for taxonomic assignment to species. A haplotype search implemented in FaBox v1.41 was used to identify and remove sequences sharing 100% nt identity [44], leaving 87 full-length genome and RT-RNase H region sequences for the analyses, respectively. Pairwise distances were determined using Standard Demarcation Tool (SDTv1.2) software [43], for 87 full-length genomes or RT-RNase H regions (1230 bp). The sequence alignments were carried out in MUSCLE [43], implemented in CLC Sequence Viewer 7.5.

Phylogenetic analysis of genomic and RT-RNase H sequences was carried out with the Maximum Likelihood (ML) algorithm available in MEGA6 [45]. Tree reconstructions used the General Time Reversible substitution model, determined by the highest Bayesian Information Criterion score obtained, with gamma distribution for invariable sites, and 1000 bootstrap iterations. Confidence values were placed at major nodes having a ≥ 70% bootstrap.

### Validation of CSSD-associated badnaviral genome sequences by sanger sequencing

To validate the de novo-assembled Illumina full-length genome sequences, six representative isolates, three each from Cote d’Ivoire and Ghana, representing Cacao vein virus (CRVV) and the established CSSV species group (four and two sequences, respectively) (Table 1), were selected for PCR amplification, cloning, and bi-directional capillary (Sanger) DNA sequencing. Purified total DNA was enriched for circular DNA by RCA. The partially enriched badnaviral DNA was used as template for PCR amplification of full-length viral genomes with sequence-specific abutting primers (Additional file 1: Table S1). Amplicons were cloned using the CloneAmp™ HiFi PCR Premix (Clonetech), according to the manufacturer’s instructions. The reaction contained 1X CloneAmp™ HiFi PCR Premix, 0.2 μM each of reverse and forward primers, 2 μL of RCA product, and nuclease-free water to a final volume of 50 μL. The PCR conditions were: denaturation at 98 °C for 2 min, followed by 40 cycles of denaturation at 98 °C for 20 s, annealing at 55 °C for 15 s, extension at 72 °C for 8 min, and final extension at 72 °C for 10 min. Amplicons were separated by agarose gel (0.8%) electrophoresis in TAE buffer, pH 8.0. Bands ~7 kbp in size were excised and gel-purified using the illustra GFX PCR and DNA Gel Band Purification kit (GE Healthcare Bio-Sciences), according to manufacturer’s instructions. The concentration of DNA was determined using the Nanodrop 2000 UV–vis spectrophotometer (Thermo Scientific). Amplicons were cloned into the pGEM5 plasmid vector (Promega), previously linearized by digestion with *Not* I (New England Biolabs). Transformation was carried out using the In-Fusion HD Cloning kit (Clontech), according to the manufacturer’s instructions. Insert size was confirmed following purification of the plasmid vector with the GeneJET Plasmid Miniprep Kit (ThermoFisher Scientific), and by restriction with *Not* I. The DNA sequence was determined for plasmids bearing a cloned insert of ≥7 kbp in size by bi-directional, capillary Sanger DNA sequencing, and primer walking (Eton Bioscience, San Diego, CA). Sequences were assembled using SeqMan Pro v.12 (DNASTAR, Madison, WI). Viral ORFs, non-coding regions, and predicted, CPDs were identified for comparison to the assembled Illumina sequences using ORF Finder, CDD, and pairwise distance analysis (SDT), as described above.

**Table 1:** Predicted conserved protein domains identified in cacao-infecting badnavirus genome sequences

Viral isolate or species	ORF1	ORF2	ORF3	ORF4	ORFX	ORFY	*Clade
CI311	DUF1319	ND	Zn, Pepsin, RT, RNase H	ND	ND	ND	I
CI135	DUF1319	ND	Zn, Pepsin, RT, RNase H	–	ND	ND	I
CI275	DUF1319	ND	Zn, Pepsin, RT, RNase H	–	–	ND	I
CIS2	DUF1319	ND	Zn, Pepsin, RT, RNase H	–	ND	ND	I
CIS3	DUF1319	ND	Zn, Pepsin, RT, RNase H	–	ND	ND	I
CI44	DUF1319	ND	Zn, Pepsin, RT, RNase H	–	ND	ND	I
GH64	DUF1319	ND	Zn, Pepsin, RT, RNase H	–	–	ND	I
GH67	DUF1319	ND	Zn, Pepsin, RT, RNase H	–	–	ND	I
GH75	DUF1319	ND	Zn, Pepsin, RT, RNase H	ND	ND	ND	I
CI134	DUF1319	ND	Zn, Pepsin, RT, RNase H	–	ND	ND	I
CI215	DUF1319	ND	Zn, Pepsin, RT, RNase H	–	ND	ND	I
CI286	DUF1319	ND	Zn, Pepsin, RT, RNase H	–	–	ND	I
CIT5	DUF1319	ND	Zn, Pepsin, RT, RNase H	–	ND	ND	I
CI301	DUF1319	ND	Zn, Pepsin, RT, RNase H	ND	ND	ND	I
TG_AJ781003	DUF1319	ND	Zn, Pepsin, RT, RNase H	–	ND	ND	I
CI_JN606110	DUF1319	ND	Pepsin, RT, RNase H	–	ND	DUF3187	I
TG_L14546	DUF1319	ND	Zn, Pepsin, RT, RNase H	–	ND	ND	I
TG_AJ534983	DUF1319	ND	Zn, Pepsin, RT, RNase H	–	ND	ND	I
GH_AJ609020	DUF1319	ND	Zn, Pepsin, RT, RNase H	–	ND	ND	I
GH_AJ609019	DUF1319	ND	Zn, Pepsin, RT, RNase H	ND	ND	ND	I
GH_AJ608931	DUF1319	ND	Zn, Pepsin, RT, RNase H	–	ND	ND	I
CaMMV	DUF1319	ND	Zn, DUF4200, Pepsin, RT, RNase H	–	–	ND	I
CYVBV	DUF1319	ND	Zn, PHD, Trim, Pepsin, RT, RNase H	–	–	ND	II

*Legend*: The West African field isolates are designated by sample code. The Genbank Accession number for each cacao-infecting badnavirus reference sequence is indicated. Predicted open reading frames (ORFs) were located using the ORF Finder tool (NCBI). The country abbreviations used are: CI-Cote d’Ivoire, GH-Ghana, and TG-Togo, for West African field isolates studied here, and available in GenBank. The conserved protein domains are indicated as: *DUF* domain of unknown function, *Zn* zinc knuckle finger, *Pepsin* pepsin-like aspartate protease, *RT* reverse transcriptase, *RNase H* ribonuclease H, *PHD* plant homeodomain finger, *Trim* trimeric-dUTPase, H, *ND* no domain predicted. The asterisk *Clade = phylogenetic clade designation based on full-length genome sequence (Fig. 3b)

## Results

### Illumina and sanger sequences

The Illumina reads were assembled into 14 full-length badnaviral genomes ranging from 6920 to 7172 bp in size. Also, BLASTn analysis against the GenBank database identified a large number of partial contigs with significant matches to CSSD-badnaviral sequences. However, in this report, only complete badnaviral genome sequences were considered, and they are derived from 410,662–31,417,994 reads with a depth of coverage of 36–6600.

PCR amplification was carried out using sequence-specific primers (Additional file 1: Table S1) and Sanger sequencing of six full-length genome sequences, representing two CSSV and three CRVV full-length genomes, and one partial genome assembled from CRVV isolate CI286. Illumina and Sanger sequence comparisons indicated that the cloned viral amplicons were nearly identical in length and sequence, at 99.0–99.9% nt identity, between the respective sequences. BLASTn analysis for the 14 apparently full-length CSSD-badnaviral genome sequences indicated the top hits in the GenBank database were consistent to previously reported CSSD-badnaviral sequences based on similarity score, sequence coverage, and e-value of zero. A comparison of the resultant Next-gen Illumina and Sanger DNA sequences using the above criteria, as well as: location of the first nt coordinate, number and location of predicted ORFs ≥10 kDa, and presence of the hallmark-badnaviral non-coding sequence region, indicated that both approaches yielded the same genome sequence. The Illumina sequences have been deposited in GenBank as the Accession numbers, KX592571-KX592584.

### Genome characterization

**Fig. 1:**
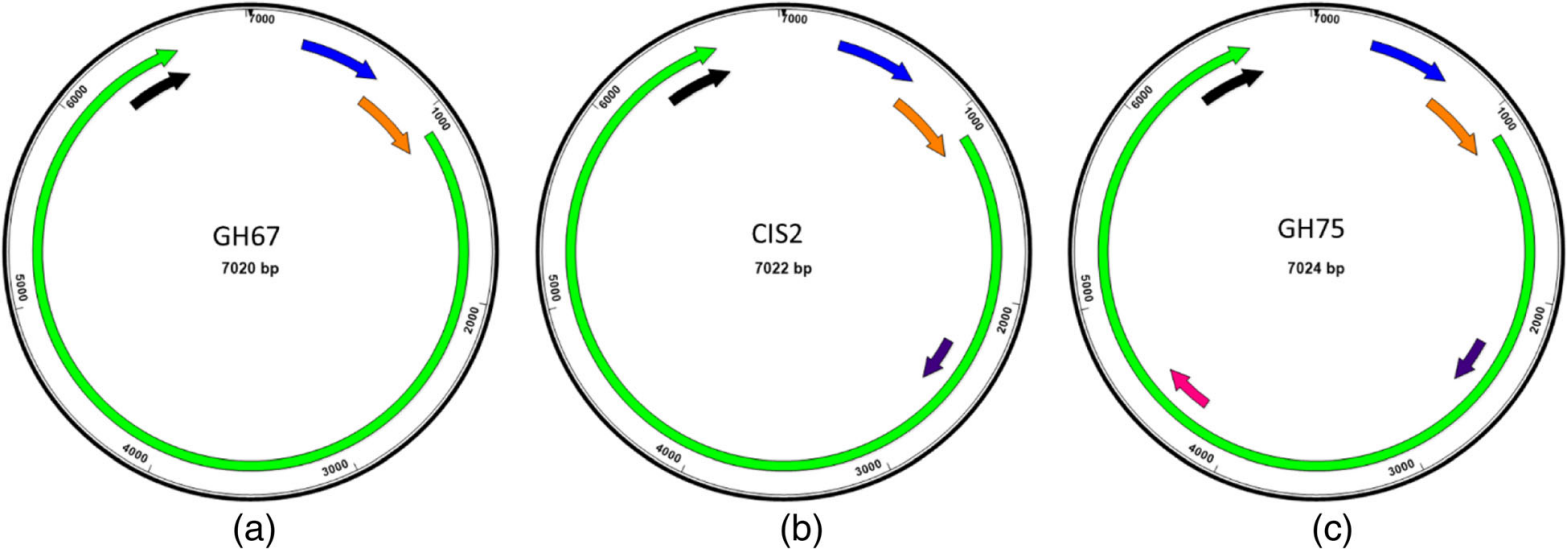
The three types of genome arrangements found among the cacao swollen shoot- badnaviral genome sequences. The predicted coding regions are indicated by filled arrows as: blue = ORF1, orange = ORF2, green = ORF3, pink = ORF4, purple = ORFX, and black = ORFY. **a** The four ORF-genome arrangement (type I), shown for isolate GH67, was also observed for isolates CI275, GH64 and CI286. **b** The five ORF-genome arrangement (type II), shown for isolate CIS2, was present among isolates CIS3, CI134, CI135, CIT5, CI44 and CI215. **c** The six ORF genome arrangement (type III), represented by isolate GH75, was present in isolates CI311 and CI301. The small black arrowhead at the top of each genome map indicates the first nucleotide coordinate, which also is the first 5′-nt of the tRNA^met^ binding site. Country name and abbreviation: CI (Cote d’Ivoire), GH (Ghana), and TG (Togo) indicating country of sample collection

Identification of predicted coding regions (>50 aa) on the CSSD-badnaviral sense strand for the 14 CSSD-genomes revealed three types of genome arrangements, arbitrarily herein referred to as I, II, and III (Fig. 1). Samples CI275, CI286, GH64, and GH67, representing isolates from Cote d’Ivoire and from Ghana, had a type I arrangement, which consisted of four predicted ORFs, 1–3 and Y (Fig. 1a). Seven isolates, CI44, CI134, CI135, CI215, CIS2, CIS3, and CIT5, all from Cote d’Ivoire, had a type II genome arrangement, comprising five predicted ORFs, 1–3, X, and Y (Fig. 1b). And, CI301, CI311, and GH75, which represent isolates from Cote d’Ivoire and Ghana, had a type III arrangement, or six ORFs 1–4, X, and Y (Fig. 1c). The CSSD-associated genome sequences each contained a predicted tRNA^met^ binding site, located at nt coordinates 1–18, at which reverse transcription is initiated [24, 46].

### Conserved protein domains

**Fig. 2:**
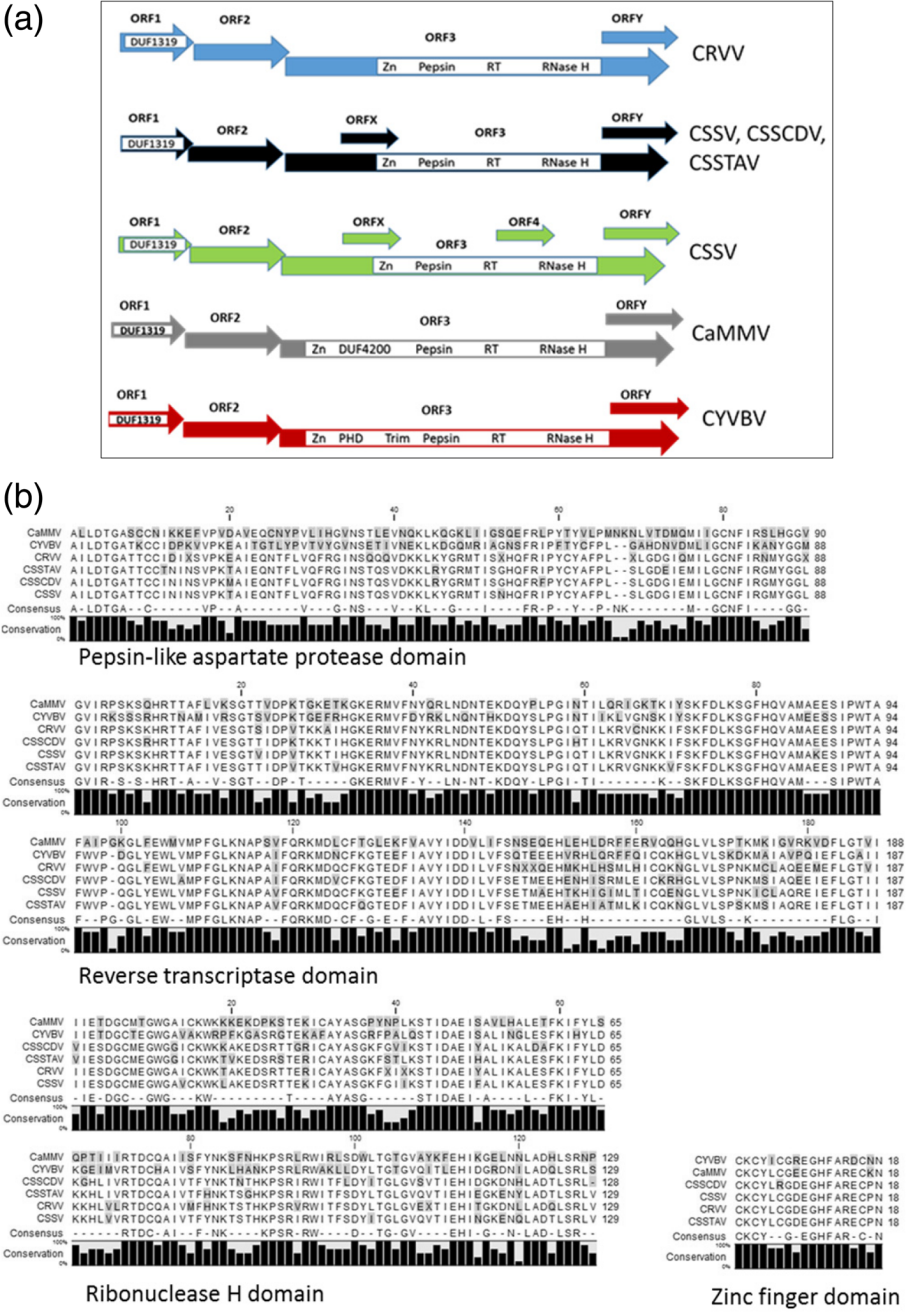
Predicted, conserved protein domains (CPDs) and corresponding amino acid sequence alignments. **a** Linear maps (not to scale) show the three known genome arrangements for the West African cacao-infecting badnaviruses, with either four (blue), five (black), or six (green) open reading frames (ORFs), compared with cacao-infecting badnaviruses from Trinidad, Cacao mild mosaic virus (CaMMV) (gray box), and Cacao yellow-vein banding virus (CYVBV) (red box), each with four ORFs. Abbreviation with virus name: CSSV = *Cacao swollen shoot virus*; CSSCDV = *Cacao swollen shoot CD virus*; CSSTAV = *Cacao swollen shoot Togo A virus*; CRVV = Cacao red vein virus; DUF = domain of unknown function; Zn = zinc knuckle finger; Pepsin = pepsin-like aspartate protease; RT = reverse transcriptase; RNase H = ribonuclease H; PHD = plant homeodomain finger; and Trim = trimeric-dUTPase. **b** Amino acid alignment of the CPDs for four representative CSSD-badnaviral genomes from West Africa, and CaMMV and CYVBV from Trinidad. Amino acid residues that differed among most or all of the cacao-infecting badnaviral isolates are shaded. The ‘consensus’ indicates the amino acids (aa) that are 100% conserved in CPDs, across the six badnaviral species, and excludes non-conserved residues, indicated by the “–“. The black vertical bars indicate the level of aa residue conservation, ranging from 0 to 100%, for the six representative genome arrangements with CPD architecture

Predicted CPD searches for the 21 genomes indicated a number of similarities, but also discernable differences, among CSSD-species and well-studied non-cacao-associated badnaviruses [10, 25, 47]. With respect to all of the CSSD-badnavirus species, CPDs were identified on ORFs 1 and 3. The ORF1 consistently harbored a domain of unknown function (DUF) 1319 [42], classified as a member of a badnavirus-specific, uncharacterized protein superfamily (Fig. 2a, Table 1). The ORF3 CPDs were identified as domains of zinc knuckle finger (Zn), pepsin-like aspartate protease (Pepsin), RT, and RNase H proteins (Table 1) present in all genomes except for CSSCDV [28], which lacked the Zn domain. Domains that were shared among all cacao-infecting badnaviruses were DUF1319 in ORF1, and Zn, Pepsin, RT and RNase H domains on ORF3. No CPDs were identified for CSSD-badnaviral species in the ORFs 2, 4, X, or Y, except for CSSCDV ORFY, which harbored a DUF3187 domain.

A survey of CPDs for the two New World cacao-infecting species, Cacao mild mosaic virus (CaMMV) and Cacao yellow vein-banding virus (CYVBV) [48], indicated that certain CPDs are conserved in ORFs 1 and 3 by all six species, while several domains were found only on ORF3 of CaMMV or CYVBV, and absent in CSSD-genomes (Table 1, Fig. 2a). In particular, DUF4200, which is unique to CaMMV ORF3, was located between the Zn and Pepsin domains. In CYVBV ORF3, the plant homeodomain finger (PHD) was identified adjacent to the Zn domain, and the trimeric-dUTPase (Trim) domain was located between the zinc finger and the aspartate protease domains (Table 1, Fig. 2a).

### Pairwise nucleotide identity for species demarcation

The SDT analysis of the RT-RNase H region identified four species based on ≥80% badnavirus species cutoff established by the International Committee on the Taxonomy of Viruses (ICTV): (i) CRVV, (ii) CSSV, (iii) *Cacao swollen shoot CD* (Cote d’Ivoire) *virus* (CSSCDV), and (iv) *Cacao swollen shoot Togo A virus* (CSSTAV) (Table 2). The best-represented species is CSSV, at 10 isolates, including those reported here and GenBank, from Ghana (AJ608931, AJ609019, and AJ609020) and Togo (AJ534983 and L14546), making it the most widely distributed species thus far. CSSCDV and CSSTAV are reported so far from Cote d’Ivoire (JN606110) and Togo (AJ781003), respectively. And, the herein proposed species, CRVV, represented by isolates from Cote d’Ivoire (CI275, CI286) and Ghana (GH64 and GH67), had the greatest extent of intraspecific divergence among the known CSSD-badnaviruses, at 28–30% (70–72% identity) (Table 3).

**Table 2:** Pairwise nucleotide identity of the RT-RNase H sequence for cacao-infecting badnavirus isolates

CSSTAV	TG_AJ781003	100																				
CSSCDV	CI_JN606110	76	100																			
	GH67	75	73	100																		
CRVV	CI286	75	72	82	100																	
	CI275	75	73	82	83	100																
	GH64	75	73	82	84	86	100															
	TG_L14546	75	75	76	75	76	76	100														
	TG_AJ534983	75	75	76	76	76	76	98	100													
	CI44	75	75	75	76	76	77	88	88	100												
	CI134	75	75	75	76	76	76	90	90	93	100											
	CI215	75	75	75	76	76	76	90	91	93	99	100										
	GH_AJ608931	75	75	75	76	75	76	90	90	92	98	98	100									
CSSV	GH75	75	75	75	76	75	76	90	90	92	98	98	99	100								
	GH_AJ609020	75	75	75	76	75	76	89	90	92	98	98	98	97	100							
	GH_AJ609019	75	75	75	76	76	76	90	90	92	98	98	98	98	98	100						
	CIS3	75	75	74	75	74	75	88	88	90	96	96	95	95	96	96	100					
	CI135	75	75	76	77	76	77	90	91	93	98	99	98	98	98	99	97	100				
	CI301	75	75	75	76	76	76	90	90	92	98	98	98	98	97	98	96	98	100			
	CI311	75	75	75	77	75	76	90	90	93	98	99	98	98	98	98	96	99	99	100		
	CIT5	75	75	76	77	76	77	90	90	93	98	99	98	98	98	98	96	99	98	99	100	
	CIS2	75	76	76	77	76	77	90	91	92	98	98	98	98	98	98	96	99	98	99	100	100

*Legend*: The sequences downloaded from GenBank are indicated by Accession number, and for each isolate, the country of origin is indicated by the country code. Each badnavirus genome sequence determined in this study is indicated by country code and sample number. The sequence alignment was carried out using MUSCLE, implemented in Standard Demarcation Tool v1.2 software, using ≥80% as the species cutoff. The abbreviations indicating the country origin of each isolate are as follows: CI-Cote d’Ivoire, GH-Ghana, and TG-Togo. Virus species abbreviations are indicated as: CSSV-*Cacao swollen shoot virus*; CSSTAV-*Cacao swollen shoot Togo A virus*; CSSCDV-*Cacao swollen shoot CD virus*; and CRVV-Cacao red vein virus

**Table 3:** Pairwise nucleotide identity for the complete genome sequence of cacao-infecting badnavirus isolates determined in this study

CRVV	CI286	100																				
	GH64	78	100																			
CRVV	CI275	78	82	100																		
	GH67	77	80	81	100																	
CSSCDV	CI_JN606110	71	71	71	71	100																
	TG_AJ781003	72	70	72	72	76	100															
	TG_L14546	71	71	72	72	75	80	100														
	TG_AJ534983	71	71	71	72	75	80	98	100													
	CI44	71	71	71	71	75	76	83	83	100												
	GH_AJ609020	72	72	71	71	75	77	85	85	89	100											
	GH75	72	71	72	71	75	77	85	85	89	97	100										
	GH_AJ608931	72	71	72	71	75	77	85	85	89	97	99	100									
CSSV	GH_AJ609019	72	71	71	71	75	77	86	85	89	97	98	98	100								
	CIS3	72	71	71	71	75	77	85	85	89	97	97	97	97	100							
	CI311	72	72	71	71	75	77	86	85	89	97	98	98	98	98	100						
	CI301	72	72	71	72	75	77	86	85	89	97	97	98	98	98	99	100					
	CI135	72	72	71	71	75	77	86	85	89	98	98	98	98	98	98	98	100				
	CIT5	72	72	71	71	75	77	86	85	90	97	98	98	98	98	98	98	98	100			
	CIS2	72	71	71	71	75	77	85	85	89	97	98	98	98	98	98	98	98	100	100		
	CI215	72	72	72	71	75	77	86	85	89	97	97	97	98	98	98	98	98	98	98	100	
	CI134	72	72	71	71	75	77	86	85	89	97	97	97	97	98	98	98	98	98	98	98	100

*Legend*: The GenBank reference sequences are indicated by Accession number, with country code. The 14 badnaviral genome sequences determined here are indicated by country code of collection and sample number. The sequence alignment was carried out using MUSCLE, implemented in the Species Demarcation Tool v1.2 software, using ≥80% as the species cutoff. The abbreviations indicating the country origin of each isolate are as follows: CI = Cote d’Ivoire, GH = Ghana, and TG = Togo. Virus species abbreviations are indicated as: CSSV = *Cacao swollen shoot virus*; CSSTAV = *Cacao swollen shoot Togo A virus*; CSSCDV = *Cacao swollen shoot CD virus*; and CRVV = Cacao red vein virus

By pairwise distance analysis of the RT-RNase H region, the 14 newly determined and seven GenBank CSSD-associated genome sequences shared 72–100% nt identity (Table 2). In contrast, the corresponding RT-RNase H region for the 21 isolates and 80 other badnaviral RT-RNase H sequences (GenBank) was 58–70% identical. Notably, CaMMV and CYVBV are as divergent from each other (40%), at 60% shared nt identity, as they are from West African CSSD-badnaviruses. Although SDT distinguished four species (≥80%) irrespective of RT-RNase H or complete viral genome sequences, species group composition was inconsistent, in particular, for the CSSD isolates. For example, SDT analysis for complete CRVV genomes (≥80% threshold) delimited CI286, and the group containing CI275, GH64, and GH67, as two groups. In contrast, the RT-RNase H analysis identified the same four isolates as one species (Tables 2 and 3). Also, the RT-RNase H analysis placed TG_AJ781003 with CSSTAV species, while by complete genome analysis, the isolate grouped with CSSV species (Tables 2 and 3). Further, the CSSTAV complete genome shared 80% nt identity with Togo isolates L14546 and AJ534983, compared to 75% for the corresponding RT-RNase H region, resulting in a conflicted species status. Finally, only CSSCDV (CI_JN606110) was delimited as the same species by both the RT-RNase H and the full-length genome sequence analysis.

### Phylogenetic analyses

**Fig. 3:**
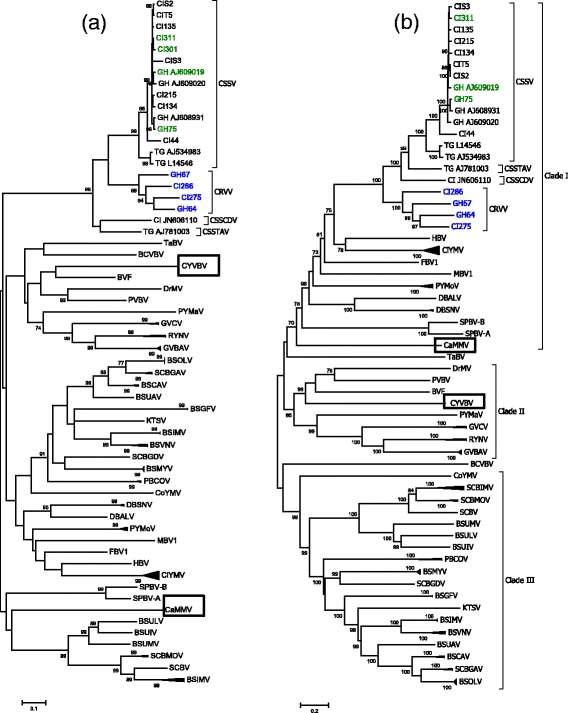
Phylogenetic analysis using Maximum likelihood (ML), implemented in MEGA 6 [45], for 87 badnaviral genomes (**a**) RT-RNase H region (1230 bp; ~coordinates 5196–6425) and (**b**) complete genome sequences. Horizontal branch lengths are proportional to genetic distance, and the values placed at major nodes indicate ≥70% bootstrap support (1000 iterations). Each genome sequence is indicated by the code for the country from which the sample was collected, and sample number. CSSD-badnavirus reference sequences are indicated by Genbank Accession number, and country code. The blue, black and green letters for the CSSD subclade isolates indicate number of coding regions per genome as: four, five and six open reading frames, respectively. Country codes and names are: CI = Cote d’Ivoire, GH = Ghana, and TG = Togo. The names of viruses are abbreviated as: PYMaV-*Pagoda yellow mosaic associated virus;* TBV-*Taro bacilliform virus*; SPBVA-*Sweetpotato badnavirus A*; SPBVB-*Sweetpotato badnavirus B*; MBV1-*Mulberry badnavirus 1*; DMV-*Dracaena mottle virus*; CoYMV-*Commelina yellow mottle virus*; BsCVBV-*Bougainvillea spectabilis chlorotic vein-banding virus*; SCBIMV-*Sugarcane bacilliform IM virus*; SCBMorV-*Sugarcane bacilliform Mor virus*; BSUMV-*Banana streak UM virus*; BSUIV-*Banana streak UI virus*; BSULV-*Banana streak UL virus*; CSSV-*Cacao swollen shoot virus*; CSSTAV-*Cacao swollen shoot Togo A virus*; CSSCDV-*Cacao swollen shoot CD virus*; CRVV-Cacao red vein virus; PYMoV-*Piper yellow mottle virus*; GVBV-*Gooseberry vein banding virus*; RYNV-*Rubus yellow net virus*; HBV-*Hibiscus bacilliform virus*; FBV1-*Fig badnavirus 1*; CiYMV-*Citrus yellow mosaic virus*; PVBV-*Pelargonium vein banding virus*; BVF-*Blackberry virus F*; PBCoV-*Pineapple bacilliform comosus virus*; GVCV-*Grapevine vein clearing virus*; BSGFV-*Banana streak GF virus*; BSMYV-*Banana streak MY virus*; DBV-*Dioscorea bacilliform virus*; SCBV-*Sugarcane bacilliform virus*; KTSV-*Kalanchoe top spotting virus*; BSUAV-*Banana streak UA virus*; BSOLV-*Banana streak OL virus*; BSCAV-*Banana streak CA virus*; BSIMV-*Banana streak IM virus*; BSVAcV-*Banana streak Vietnam Acuminata virus*, CaMMV-Cacao mild mosaic virus; and CYVBV-Cacao yellow vein-banding virus

The phylogenetic relationships were determined, based on the RT-RNase H region of 87 GenBank accessions and the 14 CSSD-badnaviral sequences. Despite the accepted use of this region for badnavirus species demarcation, the ML tree was unresolved, e.g. as a polytomy, at ≥70% bootstrap support. Within the badnavirus polytomy, the West African CSSD isolates grouped separately from the other badnaviruses, with 99% bootstrap support, indicating a close phylogenetic relationship among cacao-infecting viruses in West Africa (Fig. 3a). In contrast, ML analysis of the complete genome sequences for the same isolates (CSSD and 87 badnaviral genome sequences from GenBank), resolved three clades, each well supported by ≥70% bootstrap values. For discussion purposes here, the clades have been arbitrarily labeled as I, II and III (Fig. 3b). Clade I (70% bootstrap) contained all known (21) CSSD-associated badnaviral genomes (Fig. 3b). Clade I also contained badnaviruses associated with diverse host species, including one recently reported cacao-infecting virus from Trinidad, CaMMV [48]. A second cacao-infecting badnavirus from Trinidad, CYVBV, grouped with seven non-cacao-infecting badnaviruses in Clade II (Fig. 3b). Clade III contained 18 badnaviruses from non-cacao hosts, including the banana streak virus species group (Fig. 3b).

## Discussion

In this study, 14 full-length badnavirus genome sequences were determined by Illumina ‘virome discovery’, and confirmed by Sanger DNA sequencing of cloned, PCR amplicons. In all instances, the badnaviral genome sequences were obtained from leaf samples collected from cacao trees exhibiting foliar discoloration and swollen shoot symptoms, whereas, several of the trees also showed atypical symptoms consisting of accelerated tree decline, and rapid death. Although speculative, the latter symptoms are reminiscent of rapid necrosis and death, often associated with hypersensitive-like responses to pathogen infection.

Based on genome size and arrangement, and nt and aa sequence comparisons the genomic sequences determined here were most closely related to other previously reported badnaviruses, with the closest relatives being the three known cacao-infecting badnaviruses from West Africa: CSSV, CSSCDV, and CSSTAV [10, 25] (Additional file 2: Table S2). In addition, all of the CSSD-genomes contained the badnavirus hallmark tRNA^met^ binding site, which is reminiscent of tRNA sequences present in plant genomes [49], leading to speculation that badnaviral-associated tRNA sequences are host-derived.

The CSSD-genomes were variable with respect to predicted coding regions and genome arrangement, but were consistent among other badnaviruses, which are known to have three, four, or five predicted ORFs, variously arranged (Fig. 1). This observation has been reported previously for other CSSD isolates, which have been recently recognized as three distinct species [10]. The proposed new badnavirus species of cacao, CRVV, identified for the first time in this study, is highly divergent from CSSV, CSSDV, and CSSTSV, with which it shares only 70–72% nt identity. And, the previously known species encode five or six ORFs, compared to four ORFs predicted for the CRVV genome, indicating that CRVV is unique among the West African CSSD-badnaviruses identified so far (Additional file 2: Table S2). Compared to the four CSSD species, the New World cacao-infecting badnaviruses, CaMMV and CYVBV, described thus far encode four ORFs [48], whereas, the genomes of all known non-cacao infecting badnaviruses (genus-wide) have from three to seven ORFs.

The predicted conserved domains among the 14 genomes showed similarities to those reported in the genus *Badnavirus*, including the DUF (ORF1), and Zn, Pep, RT, and RNase H, all found in ORF3 (Table 1, Fig. 3). The only exception was the CSSCDV genome [28], which lacked a detectable Zn domain, and had an additional, unique DUF3187 domain in ORFY that is annotated as an ‘outer membrane hypothetical protein’ in certain *Proteobacteria* [42]. The absence of the Zn domain is difficult to reconcile because it has been reported to be an essential coat protein motif [25]. Interestingly, no domains have been detected in all of the CSSD-associated genomes in ORFs 4, Y, and X, including the 14 reported here (Table 1, Fig. 2). The inability to detect predicted conserved domain(s) may suggest that ‘badnavirus-novel’ domains are not necessarily absent, but that they may not be discoverable using available CDD tools [42].

A comparison between CSSD-associated genomes and the only two other cacao-infecting badnaviruses, CaMMV and CYVBV, showed interesting differences and some similarities in the CPDs. The DUF4200, which is unique to CaMMV ORF3, is annotated as a coiled-coil domain of unknown function (eukaryotic) [42]. In pararetroviruses, coiled-coil domains have been identified in virion-associated proteins, implicated in aphid transmission and for *Cauliflower mosaic virus* cell-to-cell movement [50, 51]. The PHD, unique to CYVBV ORF3, has been associated with transcriptional regulation and chromatin-associated functions [42], and the Trim also found in CYVBV ORF3, has predicted dUTPase activity e.g. catalyzes hydrolysis of dUTP-Mg complexes to dUMP and pyrophosphate [42, 52]. Although the Trim domain is unstudied for CYVBV, its presence in CYVBV and in two divergent badnaviruses, *Piper yellow mottle virus* [53] and *Dioscorea bacilliform virus* [54], suggestive of a possibly conserved, genus-wide function. The presence of the three unique domains in the two Trinidad viral genomes, and their absence in the four CSSD-associated species, make them important targets for future study.

High genomic variability was discovered among the 21 genomes from cacao, and four distinct badnavirus species were identified based on the ICTV-established ≥80% nt identity threshold on the RT-RNase H region (Table 2). Here, the previously unreported CRVV is proposed to constitute a new badnavirus species, and it is partially characterized for the first time. Until recently, all CSSD-badnaviruses were referred to as CSSV. In 2015, CSSV became recognized by the ICTV as the type species, and CSSV, CSSCDV, and CSSTAV were formally designated as distinct badnaviral species [19]. Based on these results, CRVV is the fourth cacao-infecting badnaviral species known to be endemic to West Africa. Previously, a single causal badnavirus was associated with cacao plants exhibiting different swollen shoot disease symptoms. However, the new evidence presented here shows that numerous badnaviral species and strains, evident from extensive within and between clade genomic variability, are associated with disease symptoms.

The concept of species group has not been frequently applied to plant viruses. However, a closely related ‘group of species’, banana streak virus complex, is recognized within the badnavirus genus that is reminiscent of CSSD-badnaviruses. Both comprise a group of closely related badnaviral species that are more closely related to one another than to other known badnavirus, with apparently restricted host-associations [55–57]. Similarly, the *Sugarcane mosaic virus* (*Potyviridae*) group contains closely-related, species and strains that are divergent from other known potyviruses [58]. And, within the genus, *Begomovirus* (*Geminiviridae*), five or more closely related species and multiple strains, identified thus far, incite leaf curl disease of malvaceous hosts, including the cotton crop, widespread on the Indian Subcontinent [59, 60].

Phylogenetic comparisons of all available badnaviral genome sequences, including the genome sequences determined in this study, indicated that CSSD-badnaviral genomes are more closely related to each other than to other known badnaviruses, based on the tight affiliation as a ‘sister clade’, in relation to other badnaviruses. This suggests that all CSSD-associated badnavirus species known thus far to cause swollen shoot disease share a common ancestor(s), providing robust support for West African endemism, and for the region as the center of CSSD- badnaviral diversification.

The first outbreak of CSSD was reported in Ghana [3], followed by Cote d’Ivoire ten years later. Among the available CSSV genome sequences, eleven originate from Cote d’Ivoire and three are from Ghana (Fig. 3b). The presence of CSSV in these neighboring countries is possibly suggestive of phylogeographical distribution e.g. regional CSSV endemism, however, additional sequences are required to clarify the centers of endemism of CSSV as well as the other CSSD species. Irrespective of RT-RNase H region or complete genome comparisons, CSSTAV and CSSCDV, are highly divergent from each other by 24%, and from CSSV and CRVV, by 20–25% and 28–30%, respectively (Table 3). This degree of genomic variability is suggestive of long-standing separation, and reminiscent of phylogeographical and/or host associated co-evolution. The CSSD-badnaviruses are considered endemic viruses to West Africa, and have been documented to infect many endemic tropical tree and shrub genera, including *T. cacao* relatives at the family-level, making it likely that additional undiscovered badnaviruses infect wild hosts in West Africa, with potential to undergo host shifting under opportune conditions.

Although genomic sequences are available for a relatively small number of isolates overall, the hypothesis that CRVV may have emerged in cacao as the result of a host jump from its wild reservoir(s), may be supportable based on its low shared nt identity, at ~70–72%, with other CSSD-badnavirus species extant in cacao, and could also explain why it is most closely phylogenetically-related to them. Evidence for CSSD-badnavirus infection of endemic tropical tree species is based nearly entirely on biological studies, e.g. grafting and/or mealybug transmission tests, whose results have not been verified using molecular methods. Based on these studies, a large number of CSSD hosts have been reported, including *Adansonia digitata* L., *Ceiba pentandra* L., *Cola chlamydantha* K. Schum., *Cola gigantean* A. Chev., and *Sterculia tragacantha* Lindl [61–63], and are among ~90 species in 30 plant families used as shade for cacao and other crops [64]. Molecular confirmation of suspect CSSD host-infection and accurate badnavirus identification when found, are important first steps to enabling knowledge of CSSD-badnavirus evolution and origin to be reconciled with specific epidemiological factors leading to outbreaks, to inform short-term management approaches and CSSD breeding strategies to enable sustainable production of the crop in the long term.

Historically, CSSD isolates have been characterized as ‘mild’ or ‘severe’, with mild isolates causing mild foliar symptoms that persisted for only several days, such as CSSV-N1A (AJ609020), and the characteristically prevalent, severe isolates, such as CSSV-New Juaben (AJ608931), that cause persistent foliar and shoot symptoms, decline, and tree death tree in 3–5 years [2, 65]. Although the N1A and New Juaben ‘strains’ are members of the CSSV species (Table 2), and same subclade (Fig. 3a, b), reported differences in pathogenicity are confounding. There is no definitive link between the rapid decline phenotype strains of recent, and strains previously recognized, as ‘severe’. The genetic basis for differences in pathogenicity e.g. virulence, among the CSSD-badnaviruses has not been investigated, however important clues may reside in the diverse genome arrangements and CPD architectures (Fig. 2b), particularly those associated with predicted functions in pathogenicity.

Despite nearly 100 years of CSSD research, there is no definitive understanding of the connections between origins or pathways of disease spread, or of the latter relationships with genomic diversification of cacao-infecting badnaviruses. The ‘unique to West Africa-genome type’ of the proposed CRVV species (Table 2; Fig. 3b), and its only recent association with the atypical, rapid decline and death phenotype observed in cacao trees in western Ghana during 2000, and next in eastern and then western Cote d’Ivoire by 2003, points to a possible CRVV-origin near the border of the two countries, with subsequent spread into plantations recently established in Western Cote d’Ivoire. Because CRVV-like MP sequences were obtained from symptomatic samples collected during surveys carried out in Cote d’Ivoire and Ghana in 2000–2003, [28] albeit, unknowingly, until this report, CRVV and perhaps other unknown variants may possibly have already spread to other cacao-producing countries, including Nigeria and Togo. These observations underscore the urgent need to identify the causal agent of the ‘rapid decline’ phenotype, and circumvent its further spread in cacao. This goal can only be achieved through the coordinated development and use of reliable molecular diagnostic tools, and well-supported surveillance efforts to track this dynamic badnavirus complex, and carry out epidemiological studies on a regional level in cacao plantations and in nearby suspect, endemic plant hosts of the CSSD-complex.

## Conclusions

This is the first discovery of CRVV, a previously undescribed badnavirus species infecting cacao trees in West Africa, represented thus far, by two isolates each from Ghana and Cote d’Ivoire. The CRVV-isolates were collected from locations where the first swollen shoot ‘rapid decline and death’ outbreaks were observed during 2000–2003. This is suggestive of a potential link between CRVV distribution, and the locations where the onset of rapid tree decline and death were observed within one year after foliar and swollen shoot symptom development. Although CRVV is the suspect causal agent of the rapid decline and death syndrome, Koch’s Postulates have not been proven, thus, urgent studies are now required to test this hypothesis. In addition, the phylogenetic analysis of all available cacao-associated genome sequences indicated that certain CSSV isolates in Ghana, which have been historically referred to as ‘severe strains’ of CSSV because of the debilitating symptoms they cause, are distinct from CRVV, albeit, it is important to note that CRVV and CSSV occupy an overlapping geographical range. This knowledge, together with additional, intensive CSSD surveillance using Next Generation DNA sequencing to facilitate genomic pathology studies of West African cacao plantations and suspect endemic host reservoirs, is expected to provide a data-rich resource with far-reaching applications, including the development and use of field and laboratory diagnostics to gain new insights into epidemiological and viral-host evolutionary dynamics, and provide vital assistance to breeding programs developing durable badnavirus-resistant cacao for long-term management.

## Additional files

Additional file 1: Table S1. Primers for polymerase chain reaction amplification of representative full-length badnaviral genomes associated with cacao swollen shoot disease in West Africa. The viral-specific sequences for each primer are underlined, and the first 8 or 9 bases indicate the plasmid vector sequence to which a *Not* I restriction site (indicated in *italics*) was included to facilitate cloning. The abbreviations indicating the country of sample collection, as GH = Ghana and CI = Cote d’Ivoire. (DOCX 13 kb)

Additional file 2: Table S2. The open reading frames (ORFs) predicted on the plus-strand of cacao-associated badnaviral genomes determined here, using the BLAST and ORF Finder tool algorithms (NCBI-GenBank database). BLASTn and BLASTp scores were considered for the top twenty Genbank hits. The abbreviations indicate the country of sample collection, as CI = Cote d’Ivoire, GH = Ghana, and TG = Togo. The notation of ‘ND’ denotes where no ORF was predicted. (DOCX 17 kb)

## Abbreviations

CaMMV: Cacao mild mosaic virus
CDD: Conserved domain database
CP: Coat protein
CPD: Conserved protein domain
CRVV: Cacao red vein virus
CSSCDV: *Cacao swollen shoot CD* (Cote d’Ivoire) *virus*
CSSD: Cacao swollen shoot disease
CSSTAV: *Cacao swollen shoot Togo A virus*
CSSV: *Cacao swollen shoot virus*
CYVBV: Cacao yellow vein-banding virus
DUF: Domain of unknown function
ICTV: International committee on taxonomy of viruses
ML: Maximum Likelihood
MP: Movement protein
ORF: Open reading frame
PHD: Plant homeodomain
RCA: Rolling circle amplification
RNase H: Ribonuclease H
RT: Reverse transcriptase
SDT: Sequence demarcation tool
