# Supplementary material for: The proposed new species, cacao red vein virus, and three previously recognized badnavirus species are associated with cacao swollen shoot disease
Source: Virol J. 2017 Oct 19;14:199. doi: 10.1186/s12985-017-0866-6 (PMC5649073; doi:10.1186/s12985-017-0866-6)
Supplement: Supplementary file 1 — Primers for polymerase chain reaction amplification of representative full-length badnaviral genomes associated with cacao swollen shoot disease in West Africa. The viral-specific sequences for each primer are underlined, and the first 8 or 9 bases indicate the plasmid vector sequence to which a Not I restriction site (indicated in italics) was included to facilitate cloning. The abbreviations indicating the country of sample collection, as GH = Ghana and CI = Cote d’Ivoire. (DOCX 13 kb) [file 12985_2017_866_MOESM1_ESM.docx]

**Table S1** Primers for PCR-amplification of representative full-length badnaviral genomes associated with cacao swollen shoot disease.

| **Primer name** | **Primer sequence 5’ – 3’** | **Nucleotide coordinates** |
| --- | --- | --- |
| GH67_F | ATCACTAGTGCGGCCGCAATGGCGGATGAACTATGT | 2890-2908 |
| GH67_R | GACCTGCAGGCGGCCGCGAATCCACGTTAACTT | 2874-2889 |
| GH64_F | GACCTGCAGGCGGCCGCATCACTGGACAGCAATGGT | 3858-3876 |
| GH64_R | ATCACTAGTGCGGCCGCTGGTTTTTCCTAACCAATAGG | 3837-3857 |
| GH75_F | ATCACTAGTGCGGCCGCGGATTATCCATCCAGGAG | 980-997 |
| GH75_R | GACCTGCAGGCGGCCGCTTGGAGTTTTGTGATAAG | 962-979 |
| CI44_F | ATCACTAGTGCGGCCGCGCCTATCAAGGTTAAAGCTA | 3216-3235 |
| CI44_R | GACCTGCAGGCGGCCGCCTTCCTACTTCAGG | 3202-3215 |
| CI275_F | ATCACTAGTGCGGCCGCGTTAACACCCCGAACC | 1065-1080 |
| CI275_R | GACCTGCAGGCGGCCGCTTGGCCTTTTCTTCT | 1050-1064 |
| CI286_F | GAAGCCAGTTGGTATATGTG | 1692-1711 |
| CI286_R | CTTTTGTTAAATCAATCTCC | 1672-1691 |

The virus-specific sequences contained within each primer are underlined, and the first 8-9 bases are those of the plasmid vector. A *Not* I restriction site was added (italics) to facilitate cloning. The abbreviations GH (Ghana) and CI (Cote d’Ivoire) indicate the country of sample collection.
