# Supplementary material for: The proposed new species, cacao red vein virus, and three previously recognized badnavirus species are associated with cacao swollen shoot disease
Source: Virol J. 2017 Oct 19;14:199. doi: 10.1186/s12985-017-0866-6 (PMC5649073; doi:10.1186/s12985-017-0866-6)
Supplement: Supplementary file 2 — The open reading frames (ORFs) predicted on the plus-strand of cacao-associated badnaviral genomes determined here, using the BLAST and ORF Finder tool algorithms (NCBI-GenBank database). BLASTn and BLASTp scores were considered for the top twenty Genbank hits. The abbreviations indicate the country of sample collection, as CI = Cote d’Ivoire, GH = Ghana, and TG = Togo. The notation of ‘ND’ denotes where no ORF was predicted. (DOCX 17 kb) [file 12985_2017_866_MOESM2_ESM.docx]

**Table S2** Open reading frames on the plus-strand of 14 cacao swollen shoot disease badnaviral genomes.

| Sample |  | ORF1 | ORF2 | ORF3 | ORFX | ORFY | ORF4 | Total size (bp) |
| --- | --- | --- | --- | --- | --- | --- | --- | --- |
| CI311 | Amino acid size | 143 | 145 | 1847 | 91 | 135 | 95 | 7118 |
|  | Molecular mass (kDa) | 16.78 | 15.79 | 212.18 | 10.87 | 14.54 | 11.1 |  |
|  | Nucleotide coordinates | 296-727 | 724-1161 | 1127-6670 | 2310-2585 | 6310-6717 | 4215-4502 |  |
|  | BLASTn similarity | 66-99% | 73-97% | 73-99% | 68-96% | 71-94% | 71-99% |  |
|  | BLASTp similarity | 26-99% | 28-96% | 38-98% | 54-92% | 26-93% | 98% |  |
| CI135 | Amino acid size | 143 | 145 | 1847 | 57 | 131 | ND | 7030 |
|  | Molecular mass (kDa) | 16.78 | 15.81 | 212.42 | 7 | 14.43 |  |  |
|  | Nucleotide coordinates | 296-727 | 724-1161 | 1127-6670 | 2412-2585 | 6310-6705 |  |  |
|  | BLASTn similarity | 66-99% | 74-98% | 73-99% | 71-96% | 66-96% |  |  |
|  | BLASTp similarity | 26-99% | 28-97% | 37-98% | 54-89% | 24-92% |  |  |
| CI275 | Amino acid size | 143 | 143 | 1871 | ND | 130 | ND | 7172 |
|  | Molecular mass (kDa) | 16.58 | 15.59 | 216.12 |  | 14.3 |  |  |
|  | Nucleotide coordinates | 266-697 | 694-1125 | 1088-6703 |  | 6330-6777 |  |  |
|  | BLASTn similarity | 65-75% | 66-75% | 66-76% |  | 65-68% |  |  |
|  | BLASTp similarity | 23-75% | 27-61% | 38-71% |  | 30-65% |  |  |
| CIS2 | Amino acid size | 143 | 145 | 1845 | 89 | 131 | ND | 7022 |
|  | Molecular mass (kDa) | 1677 | 15.87 | 212.42 | 10.67 | 14.3 |  |  |
|  | Nucleotide coordinates | 295-726 | 723-1160 | 1126-6663 | 2303-2572 | 6303-6698 |  |  |
|  | BLASTn similarity | 65-98% | 74-97% | 73-99% | 72-94% | 70-96% |  |  |
|  | BLASTp similarity | 25-97% | 30-97% | 38-97% | 57-91% | 29-95% |  |  |
| CIS3 | Amino acid size | 143 | 145 | 1811 | 90 | 131 | ND | 6920 |
|  | Molecular mass (kDa) | 16.78 | 15.79 | 212.45 | 10.81 | 14.3 |  |  |
|  | Nucleotide coordinates | 296-727 | 724-1161 | 1127-6560 | 2310-2582 | 6200-6595 |  |  |
|  | BLASTn similarity | 68-98% | 75-98% | 73-99% | 69-95% | 70-97% |  |  |
|  | BLASTp similarity | 26-99% | 29-98% | 38-98% | 56-90% | 30-95% |  |  |
| CI44 | Amino acid size | 143 | 145 | 1837 | 68 | 130 | ND | 7030 |
|  | Molecular mass (kDa) | 16.85 | 15.88 | 211.5 | 8.14 | 14.09 |  |  |
|  | Nucleotide coordinates | 298-729 | 726-1163 | 1129-6642 | 2372-2578 | 6285-6677 |  |  |
|  | BLASTn similarity | 66-94% | 75-87% | 73-99% | 68-84% | 69-85% |  |  |
|  | BLASTp similarity | 24-94% | 28-90% | 38-92% | 43-67% | 24-82% |  |  |
| GH64 | Amino acid size | 162 | 142 | 1879 | ND | 130 | ND | 7115 |
|  | Molecular mass (kDa) | 18.67 | 15.41 | 215.99 |  | 14.38 |  |  |
|  | Nucleotide coordinates | 215-703 | 700-1128 | 1091-6730 |  | 6361-6753 |  |  |
|  | BLASTn similarity | 71-80% | 67-84% | 66-83% |  | 64-68% |  |  |
|  | BLASTp similarity | 25-71% | 28-62% | 38-72% |  | 30-65% |  |  |
| GH67 | Amino acid size | 143 | 145 | 1841 | ND | 130 | ND | 7020 |
|  | Molecular mass (kDa) | 16.66 | 15.82 | 212.55 |  | 14.46 |  |  |
|  | Nucleotide coordinates | 276-707 | 704-1141 | 1104-6629 |  | 6260-6652 |  |  |
|  | BLASTn similarity | 69-80% | 65-92% | 65-90% |  | 65-80% |  |  |
|  | BLASTp similarity | 22-73 | 29-58% | 37-74% |  | 31-61% |  |  |
| GH75 | Amino acid size | 143 | 145 | 1847 | 91 | 131 | 99 | 7024 |
|  | Molecular mass (kDa) | 16.74 | 15.83 | 212.31 | 10.99 | 14.22 | 11.47 |  |
|  | Nucleotide coordinates | 294-725 | 722-1149 | 1125-6668 | 2308-2583 | 6308-6703 | 4213-4512 |  |
|  | BLASTn similarity | 66-99% | 74-99% | 73-99% | 69-98% | 70-98% | 71-99% |  |
|  | BLASTp similarity | 25-99% | 29-99% | 37-99% | 57-95% | 25-98% | 95% |  |
| CI134 | Amino acid size | 143 | 145 | 1846 | 90 | 131 | ND | 7012 |
|  | Molecular mass (kDa) | 16.77 | 15.82 | 212.12 | 10.82 | 14.34 |  |  |
|  | Nucleotide coordinates | 283-714 | 711-1148 | 1114-6654 | 2297-2569 | 6294-6689 |  |  |
|  | BLASTn similarity | 68-99% | 74-97% | 73-99% | 70-95% | 70-95% |  |  |
|  | BLASTp similarity | 26-97% | 29-97% | 38-98% | 60-91% | 31-93% |  |  |
| CI215 | Amino acid size | 143 | 145 | 1845 | 89 | 131 | ND | 7004 |
|  | Molecular mass (kDa) | 16.79 | 15.84 | 212.08 | 10.67 | 14.26 |  |  |
|  | Nucleotide coordinates | 278-709 | 706-1143 | 1109-6646 | 2292-2561 | 6286-6641 |  |  |
|  | BLASTn similarity | 66-98% | 74-98% | 73-99% | 71-93% | 70-95% |  |  |
|  | BLASTp similarity | 26-97% | 29-97% | 38-98% | 58-88% | 30-92% |  |  |
| CI286 | Amino acid size | 143 | 145 | 1869 | ND | 130 | ND | 7122 |
|  | Molecular mass (kDa) | 16.43 | 15.84 | 215.48 |  | 14.48 |  |  |
|  | Nucleotide coordinates | 275-706 | 703-1140 | 1103-6712 |  | 6346-6738 |  |  |
|  | BLASTn similarity | 70-80% | 65-69% | 66-96% |  | 65-67% |  |  |
|  | BLASTp similarity | 24-71% | 26-59% | 36-71% |  | 27-66% |  |  |
| CIT5 | Amino acid size | 143 | 145 | 1843 | 87 | 131 | ND | 7016 |
|  | Molecular mass (kDa) | 16.77 | 15.84 | 211.98 | 10.58 | 14.29 |  |  |
|  | Nucleotide coordinates | 295-726 | 723-1160 | 1126-6657 | 2309-2572 | 6297-6692 |  |  |
|  | BLASTn similarity | 66-97% | 74-97% | 73-99% | 72-94% | 71-97% |  |  |
|  | BLASTp similarity | 25-97% | 30-98% | 38-98% | 59-90% | 29-95% |  |  |
| CI301 | Amino acid size | 143 | 145 | 1846 | 90 | 131 | 95 | 7006 |
|  | Molecular mass (kDa) | 16.78 | 15.81 | 212.32 | 10.91 | 14.26 | 11.03 |  |
|  | Nucleotide coordinates | 296-727 | 724-1161 | 1127-6667 | 2310-2582 | 6307-6702 | 4212-4499 |  |
|  | BLASTn similarity | 66-99% | 73-97% | 73-99% | 68-96% | 71-94% | 71-99% |  |
|  | BLASTp similarity | 26-99% | 28-96% | 38-98% | 54-92% | 26-93% | 98% |  |

The ORFs were identified using the NCBI BLAST algorithm and NCBI ORF Finder tool. BLASTn and BLASTp results are based on the first 100 virus hits. The abbreviations CI (Cote d’Ivoire), GH (Ghana), and TG (Togo) indicate the country of sample collection. ND denotes where an ORF was not detected or predicted.
